# Ampicillin sulbactam impacts serum potassium level comparable to piperacillin tazobactam

**DOI:** 10.1038/s41598-025-19484-8

**Published:** 2025-10-10

**Authors:** Sho Tashiro, Ryota Tanaka, Ken Shiraiwa, Takehiro Hashimoto, Ryosuke Tatsuta, Kazufumi Hiramatsu, Hiroki Itoh

**Affiliations:** 1 Department of Clinical Pharmacy, Oita University Hospital, Yufu, Oita, 879-5593 Japan; 2 Hospital Infection Control Center, Oita University Hospital, Yufu, Oita, 879-5593 Japan

**Keywords:** Ampicillin/sulbactam, Piperacillin/tazobactam, Beta-lactam/beta-lactamase inhibitor combination, Serum potassium level, Hypokalemia, Risk factors, Infectious diseases, Adverse effects, Drug therapy

## Abstract

Ampicillin/sulbactam (ABPC/SBT) and piperacillin/tazobactam (PIPC/TAZ) are important beta-lactam/beta-lactamase inhibitor combinations. Recently, PIPC/TAZ has been reported to decrease serum potassium level (SPL). To test the hypothesis that ABPC/SBT also has the same impact, we evaluated the change in SPL by ABPC/SBT administration and compared with that by PIPC/TAZ. A retrospective, observational propensity-score matched cohort study was performed using data between 2020 and 2022. After subject selection, multiple regression analysis was conducted to identify risk factors associated with decrease in SPL. Baseline SPL, duration of ABPC/SBT or PIPC/TAZ administration, and female sex were identified as significant risk factors. Using the factors that potentially influence SPL, propensity score matching was performed, and the decrease in SPL was compared between the propensity score-matched cohorts (ABPC/SBT: n = 59, PIPC/TAZ: n = 59). The change in SPL was not significantly different between the two cohorts (− 0.28 ± 0.47 mEq/L vs. − 0.35 ± 0.51 mEq/L, *P* = 0.443). In addition, the percent decrease in SPL (10.7 ± 7.0% vs. 11.0 ± 9.7%, *P* = 0.822) and the incidence of hypokalemia (25.4% vs. 27.1%, *P* = 1.000) were not significantly different. ABPC/SBT decreases SPL comparable to PIPC/TAZ, with a clinically significant effect. Careful monitoring of SPL is necessary when patients with identified risk factors are treated with ABPC/SBT or PIPC/TAZ.

**Supplementary Information:**

The online version contains supplementary material available at 10.1038/s41598-025-19484-8.

## Introduction

Beta-lactam/beta-lactamase inhibitor combinations (BL/BLI) are clinically important antibacterial agents for various moderate-to-severe infections. These agents possess broad-spectrum antibacterial activities owing to the combined actions of the key active ingredients: the beta-lactam antibiotics and the beta-lactamase inhibitors as the suicide substrate. Beta-lactamase inhibitors circumvent antibiotic resistance mediated by beta-lactamases to broaden the spectrum of antibacterial activity^1,2^. In clinical settings, ampicillin/sulbactam (ABPC/SBT) and piperacillin/tazobactam (PIPC/TAZ) are frequently administered to hospitalized patients worldwide. The safety profiles of these agents have been reported based on accumulated clinical data, and the reported adverse events include shock, anaphylaxis, skin lesions, diarrhea, delirium, liver damage, and kidney injury^3–7^.

Recently, the safety of PIPC/TAZ has received considerable attention. It has been revealed that PIPC/TAZ decreases serum potassium level (SPL) and induces hypokalemia. Several studies have reported high incidence of hypokalemia of approximately 15–25%, and that hypokalemia often occurs 2–3 days after the start of administration^8–11^. The incidence of hypokalemia has been described in the package insert of Zosyn® in various countries^12,13^. On the other hand, there is a paucity of available data on ABPC/SBT^5,14^. Since severe hypokalemia may lead to life-threatening events^15–17^, elucidation of the safety profile of ABPC/SBT is urgently needed and would contribute to safer use.

We hypothesized that ABPC/SBT decreases SPL and induces hypokalemia similar to PIPC/TAZ, because these agents are both BL/BLI and the two beta-lactam antibiotics have similar chemical structures^18^. To test this hypothesis, we carried out a single-center, retrospective, propensity score-matched, observational cohort study. The present study has three main objectives: to identify the risk factors associated with the decrease in SPL during administration of BL/BLI, to compare the decrease in SPL between ABPC/SBT and PIPC/TAZ, and to determine the safety profile of ABPC/SBT using PIPC/TAZ as a positive control.

## Methods

### Study design and subjects

A single-center, retrospective, propensity score-matched observational cohort study was performed based on medical records for three years (between January 1, 2020 and December 31, 2022) at Oita University Hospital (Yufu, Oita, Japan). Hospitalized patients who received intravenous ABPC/SBT or PIPC/TAZ for more than three days were included. Exclusion criteria were as follows: patients without sufficient data on background characteristics, clinical laboratory test values, and information on administered drugs (If data of even one of the items in Table 1 could not be collected within 48 h of the start of BL/BLI administration, the patient was excluded.); patients with hypokalemia (SPL < 3.5 mEq/L) or hyperkalemia (SPL > 5.5 mEq/L)^9,15^ when BL/BLI was initiated; patients aged < 18 years; and patients on dialysis. In each cohort, only first encounters were included.

**Table 1:** Patient characteristics, baseline clinical laboratory test values, and information on administered drugs of the two cohorts before propensity score matching.

	ABPC/SBT cohort (n = 191)	PIPC/TAZ cohort (n = 63)	*P*-value
Sex (male/female)	142/49	37/26	0.025
Age (median [IQR])	67.0 (54.0–77.5)	76.0 (65.5–84.0)	< 0.001
BMI (kg/m^2^, mean ± SD)	22.9 ± 4.6	22.5 ± 5.0	0.671
eGFR (mL/min/1.73 m^2^, mean ± SD)	72.9 ± 30.6	53.8 ± 32.0	< 0.001
ALT (U/L, median [IQR])	17.1 (12.2–32.1)	13.7 (9.6–40.6)	0.366
AST (U/L, median [IQR])	20.2 (15.6–31.9)	21.7 (16.3–42.3)	0.181
ALB (g/dL, mean ± SD)	3.4 ± 0.8	2.7 ± 0.5	< 0.001
Serum sodium level (mEq/L, median [IQR])	138.0 (135.8–139.5)	136.6 (134.0–138.8)	0.008
SPL (mEq/L, median [IQR])	4.0 (3.8–4.4)	4.0 (3.8–4.4)	0.826
Duration of BL/BLI administration (days, median [IQR])	7.0 (5.0–9.5)	6.0 (4.5–9.5)	0.339
BL/BLI prescribed daily dose/defined daily dose* (mean ± SD)	0.73 ± 0.25	0.65 ± 0.21	0.012
Patients administered drugs that potentially increase SPL	174 (91.1%)	61 (96.8%)	0.172
Infusion including potassium	143	54	
Potassium supplement	20	14	
NSAIDs	96	26	
ARBs	40	9	
ACE-Is	11	5	
Beta-blockers	30	14	
Potassium-sparing diuretics	14	7	
ST	6	6	
Digoxin	2	0	
Tacrolimus	2	5	
Patients administered drugs that potentially decrease SPL	150 (78.5%)	49 (77.8%)	1.000
Diuretics (except potassium-sparing diuretics alone)	38	25	
Mineralocorticoids and glucocorticoids	99	22	
Amphotericin B	0	1	
Anticancer drugs**	1	0	
Laxatives and enemas	58	20	
β_2_ agonist	19	6	
Xanthines	1	0	
Insulin	19	14	
Potassium- scavenger	3	2	
Potassium load from concomitant drugs during BL/BLI administration (mEq/day, median [IQR])	5.5 (0.4–12.2)	8.3 (2.4–20.0)	0.023

BL/BLI, beta-lactam/beta-lactamase inhibitor combination; ABPC/SBT, ampicillin/sulbactam; PIPC/TAZ, piperacillin/tazobactam; BMI, body mass index; eGFR, standardized estimated glomerular filtration rate; ALT, alanine aminotransferase; AST, aspartate aminotransferase; ALB, serum albumin; SPL, serum potassium level; NSAIDs, non-steroidal anti-inflammatory drugs; ARBs, angiotensin II receptor blockers; ACE-Is, angiotensin-converting enzyme inhibitors; ST, sulfamethoxazole/trimethoprim; ARNI, angiotensin receptor-neprilysin inhibitor.

*WHO Collaborating Centre for Drug Statistics Methodology: ATC/DDD Index 2025. [cited 2025 May 20] https://atcddd.fhi.no/atc_ddd_index/

**Anticancer drugs included cisplatin and other platinum drugs, cyclophosphamide, ifosfamide, cetuximab, panitumumab, temsirolimus, methotrexate, abiraterone, and eribulin.

### Outcome definition

The primary endpoint was the change in SPL during BL/BLI administration, defined as the lowest SPL during BL/BLI administration minus baseline SPL. Secondary endpoints were the percent decrease in SPL and the incidence of hypokalemia during BL/BLI administration. The percent decrease in SPL was calculated by the following equation: change in SPL during BL/BLI administration/baseline SPL × 100. The incidence of hypokalemia was calculated as follows: number of patients with hypokalemia/number of all patients administered BL/BLI × 100. The incidence of hypokalemia within 3, 5, 7, and 14 days after starting BL/BLI administration, and all periods during BL/BLI administration was calculated. Hypokalemia was defined as SPL < 3.5 mEq/L, according to previous reports^9,10^.

### Data collection

Patient characteristics at baseline, clinical laboratory test values, and information on administered drugs were extracted from the electronic medical records. Patient characteristics included sex (male or female), age, height, and body weight. Clinical laboratory test values included alanine aminotransferase, aspartate aminotransferase, serum albumin (ALB), standardized estimated glomerular filtration rate (mL/min/1.73 m^2^) (eGFR), SPL, and serum sodium level. All SPL levels during BL/BLI administration on patients meeting inclusion criteria were extracted from the electronic record in order to calculate the changes in SPL. The information on administered drugs included duration of BL/BLI administration, total dosage of BL/BLI, concomitant drugs during BL/BLI administration, and potassium load from concomitant drugs (potassium infusion and potassium supplements) during BL/BLI administration.

### Statistical analyses

Statistical analyses were carried out using EZR (Saitama Medical Center, Jichi Medical University, Saitama, Japan), a graphical user interface for R (R Foundation for Statistical Computing, Vienna, Austria). Data of baseline patient characteristics, clinical laboratory test values, and information on administered drugs are expressed as number (%) for categorical variables, and mean ± standard deviation or median [interquartile range] for continuous variables. Categorical variables were analyzed using Fisher’s exact test. All data of continuous variables were tested for normal distribution by Kolmogorov–Smirnov test. Data that were normally distributed were expressed as mean ± standard deviation and analyzed by t-test. Data that were not normally distributed were expressed as median [interquartile range] and analyzed by Mann–Whitney U-test. The Kaplan–Meier survival curve was analyzed by log-lank test. A *P*-value < 0.05 was deemed to indicate a statistically significant difference.

Subjects meeting the selection criteria were classified into the ABPC/SBT and PIPC/TAZ cohorts. Multiple regression analysis was performed using data from all selected subjects to determine the risk factors associated with the decrease in SPL during BL/BLI administration. For analysis of the primary endpoint, the dependent variable was change in SPL during BL/BLI administration, and the following factors were selected as independent variables based on clinical relevance: sex, age, body mass index (BMI), concomitant use of drugs that potentially decrease SPL^9,10^, eGFR, baseline SPL, type of BL/BLI (ABPC/SBT or PIPC/TAZ), and duration of BL/BLI administration. Multiple regression equation was evaluated using *P*-value, adjusted R square, and all variance inflation factors.

One-to-one propensity score matching was performed to adjust for the differences in covariates at baseline between the ABPC/SBT and PIPC/TAZ cohorts, and the propensity score-matched cohorts were used in comparisons of primary and secondary outcomes. Propensity scores were estimated by a logistic regression model using EZR. Using the nearest neighbor matching method, one patient in one cohort was matched to one patient in the other cohort based on estimated propensity scores with the caliper width set at 0.2 standard deviations without replacement^19,20^. The following factors that potentially influence SPL were adopted as independent variables when estimating propensity scores: sex, age, BMI, eGFR, concomitant use of drugs that potentially decrease or increase SPL^9,10^, baseline SPL, duration of BL/BLI administration, and potassium load from concomitant drugs during BL/BLI administration (mEq/day). After propensity score matching, primary and secondary endpoints were compared between the two adjusted cohorts.

### Ethical consideration

This study was performed after approval by the Ethics Committee of Oita University Faculty of Medicine (Review reference number: 2731). Due to the retrospective nature of the study, the need to obtain the informed consent was waived by the Ethics Committee of Oita University Faculty of Medicine. In addition, all research was performed in accordance with the Declaration of Helsinki.

## Results

### Demographic and clinical data before propensity score matching

**Fig. 1:**
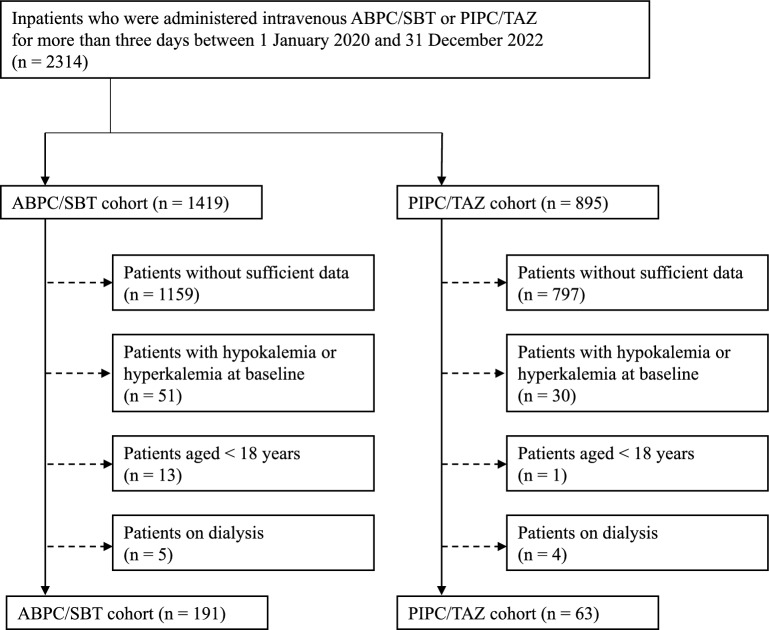
Flowchart of patient selection. ABPC/SBT, Ampicillin/sulbactam; PIPC/TAZ, piperacillin/tazobactam.

A total of 2314 patients were extracted from the medical record review based on the inclusion criteria (Fig. 1). The patients were divided into the ABPC/SBT cohort (n = 1419) and the PIPC/TAZ cohort (n = 895). After selection according to the exclusion criteria, 254 patients [ABPC/SBT cohort (n = 191) and PIPC/TAZ cohort (n = 63)] were finally included in the analyses.

Table 1 displays patient characteristics, baseline clinical laboratory test values, and information on administered drugs of the two cohorts before propensity score matching. Sex, age, eGFR, ALB, serum sodium level, daily dose, and potassium load from concomitant drugs during BL/BLI administration were significantly different between the two cohorts. These differences may suggest that more complicated and severe patients were treated with PIPC/TAZ. However, BL/BLI prescribed daily dose/defined daily dose was not significantly different between the two cohorts.

### Risk factors associated with SPL decrease during BL/BLI administration in unadjusted cohort

Multiple regression analysis of risk factors associated with the decrease in SPL during BL/BLI administration was conducted in all selected patients (n = 254) before propensity score matching (Table 2). The following three risk factors were identified to be independently associated with the decrease in SPL during BL/BLI administration: sex (female) [(*P* = 0.008, standardized partial regression coefficient (β) =  − 0.143], baseline SPL (higher) (*P* < 0.001, β =  − 0.476), and duration of BL/BLI administration (longer) (*P* < 0.001, β =  − 0.234). The type of BL/BLI (ABPC/SBT or PIPC/TAZ) was not a significant risk factor (*P* = 0.177, β = 0.075). The *P*-value of the multiple regression equation was < 0.01 and the adjusted R square was 0.307. In addition, all variance inflation factors were below 1.28, suggesting negligible multicollinearity among predictors.

**Table 2:** Multiple regression analysis of risk factors associated with the decrease in SPL during BL/BLI administration in all selected patients (n = 254) before propensity score matching.

Covariate	*P*-value	Standardized partial regression coefficient (β)	Coefficients (B)	
			Estimate	Standard error
Sex (male = 0, female = 1)	0.008	− 0.143	− 0.150	0.056
Age	0.511	− 0.039	− 0.001	0.002
BMI (kg/m^2^)	0.155	0.077	0.008	0.006
Concomitant use of drugs that potentially decrease SPL (Yes = 0, No = 1)	0.482	− 0.038	− 0.044	0.062
eGFR	0.268	0.066	0.001	0.001
Baseline SPL	< 0.001	− 0.476	− 0.537	0.060
Type of BL/BLI (PIPC/TAZ = 0, ABPC/SBT = 1)	0.177	0.075	0.083	0.062
Duration of BL/BLI administration	< 0.001	− 0.234	− 0.019	0.004

BL/BLI, beta-lactam/beta-lactamase inhibitor combination; ABPC/SBT, ampicillin/sulbactam; PIPC/TAZ, piperacillin/tazobactam; SPL, serum potassium level; BMI, body mass index; eGFR, standardized estimated glomerular filtration rate; PIPC/TAZ, piperacillin/tazobactam; ABPC/SBT, ampicillin/sulbactam.

### Distribution of days to hypokalemia onset after start of BL/BLI administration

Figure S1 shows the distribution of days to hypokalemia onset after the initiation of BL/BLI administration in patients who developed hypokalemia (40 in the SBT/ABPC cohort, 17 in the TAZ/PIPC cohort) among all the selected patients. The median [interquartile range] duration to hypokalemia onset was 4.0 [3.0–7.3] days in the SBT/ABPC cohort and 4.0 [3.0–10.0] days in the TAZ/PIPC cohort, with no significant difference between the two cohorts (*P* = 0.902). Approximately 70% of the hypokalemia cases had onset within five days of starting antibiotics, and all hypokalemia cases occurred within 30 days.

Figure S2 shows the Kaplan–Meier survival curves of the SBT/ABPC cohort and the TAZ/PIPC cohort for onset of hypokalemia. No significant difference was detected between the curves (*P* = 0.158).

### Comparison of outcomes between ABPC/SBT and PIPC/TAZ cohorts after propensity score matching

Fifty-nine patients in the ABPC/SBT cohort were matched with 59 patients in the PIPC/TAZ cohort using propensity scores. Patient characteristics, clinical laboratory test values, and information on administered drugs in the propensity score-matched cohorts are summarized in Table 3. After matching, the two cohorts had no significant differences in patient background and baseline clinical parameters, aside for ALB (3.2 ± 0.6 g/dL vs. 2.7 ± 0.5 g/dL, *P* < 0.001) and serum sodium level (138.0 ± 4.7 mEq/L vs. 136.1 ± 3.9 mEq/L, *P* = 0.020).

**Table 3:** Patient characteristics, baseline clinical laboratory test values, and information on administered drugs in the two propensity score-matched cohorts.

	Propensity score-matched		
	ABPC/SBT cohort (n = 59)	PIPC/TAZ cohort (n = 59)	*P*-value
Sex* (male/female)	38/21	37/22	1.000
Age*	74.9 ± 10.9	73.0 ± 14.4	0.430
BMI* (kg/m^2^)	23.6 ± 5.7	22.8 ± 5.0	0.403
eGFR* (mL/min/1.73 m^2^)	57.1 ± 28.4	54.4 ± 32.5	0.627
ALT (U/L)	16.4 (11.2–27.0)	13.7 (9.6–40.6)	0.925
AST (U/L)	20.5 (16.5–33.7)	21.7 (16.3–45.3)	0.559
ALB (g/dL)	3.2 ± 0.6	2.7 ± 0.5	< 0.001
Serum sodium level (mEq/L)	138.0 ± 4.7	136.1 ± 3.9	0.020
SPL* (mEq/L)	4.1 ± 0.4	4.1 ± 0.5	0.869
Duration of BL/BLI administration (day)*	7.0 (5.0–10.0)	6.0 (4.5–9.5)	0.425
BL/BLI prescribed daily dose/defined daily dose**	0.70 ± 0.25	0.64 ± 0.21	0.211
Patients administered drugs that potentially increase SPL*	57	57	1.000
Infusion including potassium	48	50	
Potassium supplement	11	12	
NSAIDs	27	26	
ARBs	18	8	
ACE-Is	6	5	
Beta-blockers	16	13	
Potassium-sparing diuretics	7	6	
ST	2	6	
Digoxin	2	0	
Tacrolimus	0	4	
Patients administered drugs that potentially decrease SPL*	46	46	1.000
Diuretics (except potassium-sparing diuretics alone)	18	23	
Mineralocorticoids and glucocorticoids	23	20	
Amphotericin B	0	1	
Anticancer drugs***	0	0	
Laxatives and enemas	21	20	
β_2_ agonist	8	5	
Xanthines	0	0	
Insulin	11	13	
Potassium scavenger	2	2	
Potassium load from concomitant drugs during BL/BLI administration (mEq/day)*	10.0 (4.1–20.9)	8.0 (2.0–20.0)	0.624

BL/BLI, beta-lactam/beta-lactamase inhibitor combination; ABPC/SBT, ampicillin/sulbactam; PIPC/TAZ, piperacillin/tazobactam; BMI, body mass index; eGFR, standardized estimated glomerular filtration rate; ALT, alanine aminotransferase; AST, aspartate aminotransferase; ALB, serum albumin; SPL, serum potassium level; NSAIDs, non-steroidal anti-inflammatory drugs; ARBs, angiotensin II receptor blockers; ACE-Is, angiotensin-converting enzyme inhibitors; ST, sulfamethoxazole/trimethoprim; ARNI, angiotensin receptor-neprilysin inhibitor.

*Indicates factors used for propensity score matching.

**WHO Collaborating Centre for Drug Statistics Methodology: ATC/DDD Index 2025. [cited 2025 May 20] https://atcddd.fhi.no/atc_ddd_index/

***Anticancer drugs included cisplatin and other platinum drugs, cyclophosphamide, ifosfamide, cetuximab, panitumumab, temsirolimus, methotrexate, abiraterone, and eribulin.

Both ABPC/SBT and PIPC/TAZ decreased SPL, and the change in SPL was not significantly different between the ABPC/SBT (−0.28 ± 0.47 mEq/L) and the PIPC/TAZ (−0.35 ± 0.51 mEq/L) cohorts (*P* = 0.443) (Table 4). The percent decrease in SPL was also not significantly different between the ABPC/SBT (10.7 ± 7.0%) and the PIPC/TAZ (11.0 ± 9.7%) cohorts (*P* = 0.822). In addition, the incidence of hypokalemia during BL/BLI administration was 25.4% (15/59) in the ABPC/SBT cohort and 27.1% (16/59) in the PIPC/TAZ cohort, also with no significant difference between the two cohorts (*P* = 1.000, odds ratio = 1.091, 95% confidence interval = 0.443–2.696). The incidence of hypokalemia for the ABPC/SBT cohort vs. the PIPC/TAZ cohort within 3, 5, 7, and 14 days after starting BL/BLI administration was 3.4% vs. 8.5% (*P* = 0.439), 18.6% vs. 16.9% (*P* = 1.000), 20.3% vs. 18.6% (*P* = 1.000), and 23.7% vs. 23.7% (*P* = 1.000), respectively, showing no significant differences between the two cohorts.

**Table 4:** Comparison of outcomes of BL/BLI administration between propensity score-matched ABPC/SBT and PIPC/TAZ cohorts.

	Propensity score-matched		
	ABPC/SBT cohort (n = 59)	PIPC/TAZ cohort (n = 59)	Result of analysis
Change in SPL (mEq/L)	− 0.28 ± 0.47	− 0.35 ± 0.51	*P* = 0.443
Percent decrease in SPL (%)	10.7 ± 7.0	11.0 ± 9.7	*P* = 0.822
Incidence of hypokalemia during BL/BLI administration (%)	25.4	27.1	*P* = 1.000, OR = 1.091, 95%CI = 0.443–2.696
Incidence of hypokalemia within 3 days after starting administration (%)	3.4	8.5	*P* = 0.439, OR = 0.382, 95%CI = 0.035–2.453
Incidence of hypokalemia within 5 days after starting administration (%)	18.6	16.9	*P* = 1.000 OR = 1.122, 95%CI = 0.392–3.249
Incidence of hypokalemia within 7 days after starting administration (%)	20.3	18.6	*P* = 1.000, OR = 1.113, 95%CI = 0.405–3.090
Incidence of hypokalemia within 14 days after starting administration (%)	23.7	23.7	*P* = 1.000, OR = 1.000, 95%CI = 0.392–2.554

BL/BLI, beta-lactam/beta-lactamase inhibitor combination; ABPC/SBT, ampicillin/sulbactam; PIPC/TAZ, piperacillin/tazobactam; SPL, serum potassium level; OR, odds ratio; 95%CI, 95% confidence interval.

## Discussion

The present study identified the risk factors associated with the decrease in SPL during BL/BLI administration, and revealed that ABPC/SBT also decreased SPL and induced hypokalemia comparable to PIPC/TAZ. To the best of our knowledge, this is the first report to demonstrate the decrease in SPL by administration of ABPC/SBT. These findings are important for safe administration of ABPC/SBT and PIPC/TAZ.

It is clinically important to know the risk factors associated with the decrease in SPL during BL/BLI administration, as well as the frequent timeframe of hypokalemia occurrence. This study identified three risk factors: baseline SPL, duration of BL/BLI administration, and female sex (Table 2). Of these three factors, duration of administration and female sex have been reported previously^9,10,21^. Potassium is mainly stored in muscle mass, and the potassium capacity is important to resist changes in SPL^15^. Since the muscle mass of women and older individuals is generally lower than that of men and younger individuals, women and older age have been reported to be risk factors for hypokalemia^21,22^. However, older age was not identified as a risk factor in our study, which is inconsistent with previous reports^9,10^. In the present study, the patients analyzed were probably older than the subjects in previous reports, and the number of younger patients was not sufficiently large. Moreover, the most frequent time of hypokalemia occurrence was within five days after start of administration (Fig. S1), which is also close to the findings in previous reports^9,10^. These findings suggest that monitoring SPL early after initiation of administration is desirable.

Recently, a higher incidence of hypokalemia caused by PIPC/TAZ has been reported, including 13.9% by Seo et al.^9^, 24.8% by Kuramoto et al.^10^, and 12.6% by Kaya et al.^11^. We found that the mean (± standard deviation) change in SPL was -0.35 ± 0.51 mEq/L and the incidence of hypokalemia was 27.1% in the propensity score-matched PIPC/TAZ cohort (Table 4), which was slightly higher than previous reports. This could be explained by the following two reasons. First, previous studies excluded patients who were administered drugs that potentially decrease SPL^9,10^, whereas those patients were not excluded in this study. The reason is that most of the enrolled patients (approximately 80%) received these types of drugs in our institute, excluding these patients would pose difficulty in collecting a statistically sufficient number of subjects. Second, the patients analyzed were probably older (74.9 ± 10.9 years in the ABPC/SBT cohort, 73.0 ± 14.4 years in the PIPC/TAZ cohort) than the subjects in previous studies, although older age was not identified as a risk factor in our study.

Using the validated PIPC/TAZ cohort as positive control for hypokalemia and comparing two cohorts with balanced covariates after propensity score matching, the most striking finding of this study is that ABPC/SBT decreases SPL (−0.28 ± 0.47 mEq/L) and induces hypokalemia (25.4%) comparable to PIPC/TAZ (Table 4). In addition, the type of BL/BLI (ABPC/SBT or PIPC/TAZ) was not a significant risk factor in the multiple regression analysis (Table 2). The mechanism of development of hypokalemia due to PIPC/TAZ has been discussed in several studies. In the kidney, penicillins are thought to act as unabsorbed anions in the cortical collecting tubules, promoting compensatory potassium excretion into the urine. This chemical property of penicillins together with the body’s response led to hypokalemia^16,17,23,24^. Given the similar chemical structure and pKa between ABPC and PIPC, ABPC may also act in the same manner as PIPC. Collectively, these facts support the findings of this study. PIPC/TAZ decreased SPL slightly more than ABPC/SBT (Table 4), which may reflect that a single dose of PIPC/TAZ is generally larger than that of ABPC/SBT. Moreover, the beta-lactamase inhibitors (SBT and TAZ) could also act as unabsorbed anions, given the similar structures with penicillins^18^, and pKa (approximately 2.6 for both) described in the interview form of Unasyn® and Zosyn®. These inhibitors might be additional promotors of potassium excretion. Hence, the decrease in SPL due to ABPC/SBT or PIPC/TAZ is possibly greater compared with penicillins alone (such as penicillin G, ABPC and PIPC). Further studies are needed to investigate the above mechanism.

In the present study, the change in SPL during BL/BLI administration was −0.28 ± 0.47 mEq/L in the ABPC/SBT cohort and −0.35 ± 0.51 mEq/L in the PIPC/TAZ cohort (Table 4). This change in SPL was calculated in all patients (with and without hypokalemia). Thus, the magnitude of the change was relatively small. However, in a sub-group analysis in which change in SPL was calculated only in patients with hypokalemia, the change in SPL was − 0.51 ± 0.27 mEq/L in the ABPC/SBT cohort and -0.79 ± 0.41 mEq/L in the PIPC/TAZ cohort (*P* = 0.040). These changes are comparable to those in a previous report^10^. In addition, the incidence of hypokalemia within 5 days after starting administration was approximately 20% (Table 4), which would support a clinically significant decrease in SPL after BL/BLI administration. The incidence of hypokalemia was similar to that of other drugs (liposomal-amphotericin B, 23.1%^10^; high-dose furosemide, 43.1%^25^).

This study has some limitations. First, as aforementioned, patients receiving drugs that potentially influence SPL were not excluded. Except for potassium infusion and potassium supplements, it was not possible to quantify the effect of those drugs on SPL. Second, this is a single-center, retrospective, propensity score-matched, observational cohort study; therefore, unknown confounding factors may affect the results, and selection bias may exist. Third, the clinical consequences of hypokalemia, such as ECG changes, were not evaluated. Fourth, it was not possible to determine the number of patients who received potassium supplements for the management of hypokalemia occurring during BL/BLI administration. Lastly, the type of infection and patient location at baseline were not clear, although they would affect the SPL^26,27^.

## Conclusion

This study revealed that ABPC/SBT decreased SPL and induced hypokalemia comparable to PIPC/TAZ. Analysis of the propensity score-matched cohorts showed high incidence of hypokalemia of approximately 30%. In addition, baseline SPL, duration of BL/BLI administration, and female sex were identified as risk factors associated with the decrease in SPL. Monitoring SPL is necessary when administering ABPC/SBT or PIPC/TAZ, especially in patients with identified risk factors. These findings would contribute to safe use of BL/BLI.

## Supplementary Information

Supplementary Information 1.

Supplementary Information 2.

## Data Availability

The datasets used and/or analyzed during the current study are available from the corresponding author on reasonable request.
