# Supplementary material for: Ampicillin sulbactam impacts serum potassium level comparable to piperacillin tazobactam
Source: Sci Rep. 2025 Oct 10;15:35517. doi: 10.1038/s41598-025-19484-8 (PMC12514168; doi:10.1038/s41598-025-19484-8)
Supplement: Supplementary file 1 — Supplementary Information 1. [file 41598_2025_19484_MOESM1_ESM.pptx]

## Slide 1
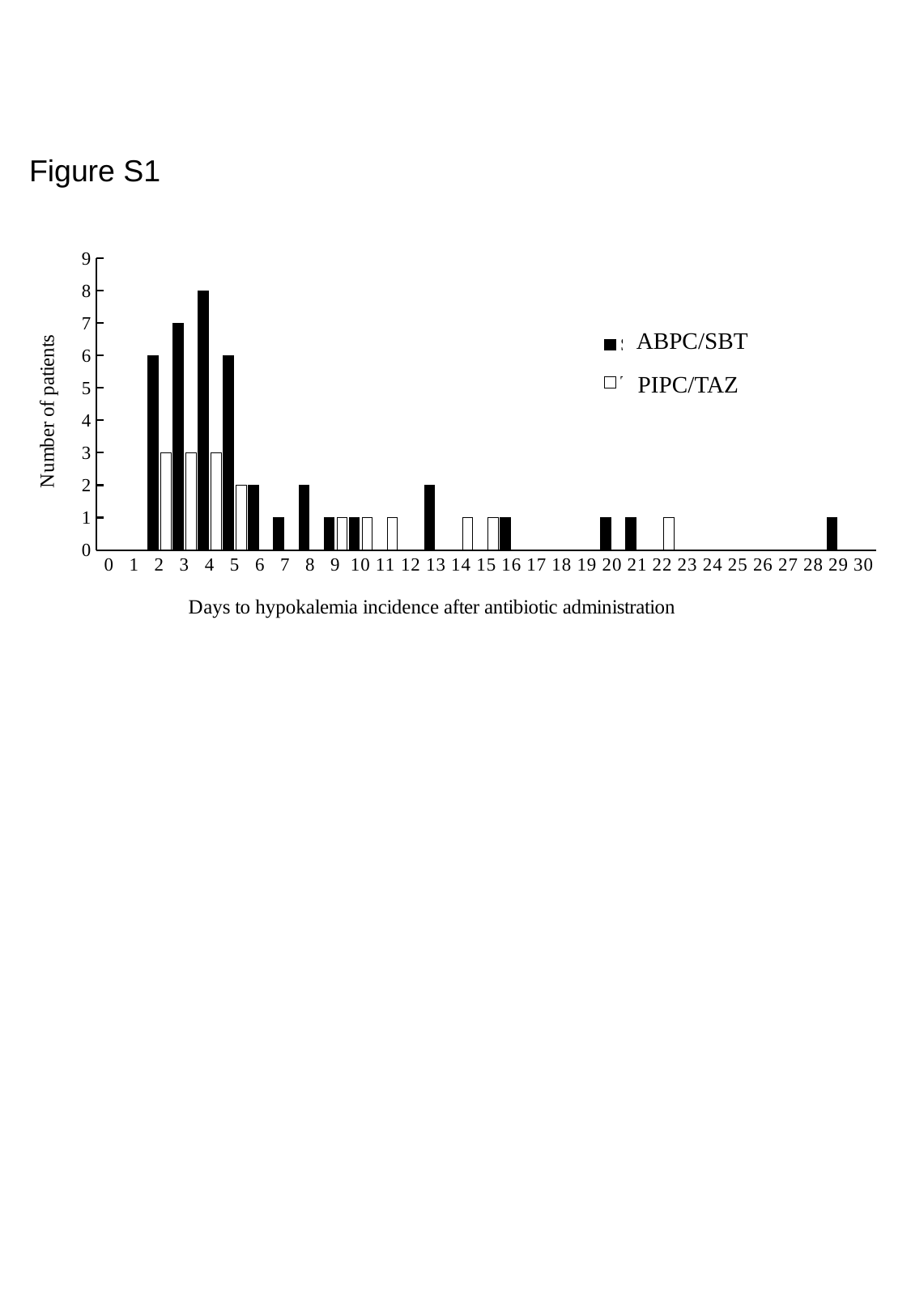

Figure S1
### Chart
| Category | | |
|---|---|---|
| 0 | 0.0 | 0.0 |
| 1 | 0.0 | 0.0 |
| 2 | 6.0 | 3.0 |
| 3 | 7.0 | 3.0 |
| 4 | 8.0 | 3.0 |
| 5 | 6.0 | 2.0 |
| 6 | 2.0 | 0.0 |
| 7 | 1.0 | 0.0 |
| 8 | 2.0 | 0.0 |
| 9 | 1.0 | 1.0 |
| 10 | 1.0 | 1.0 |
| 11 | 0.0 | 1.0 |
| 12 | 0.0 | 0.0 |
| 13 | 2.0 | 0.0 |
| 14 | 0.0 | 1.0 |
| 15 | 0.0 | 1.0 |
| 16 | 1.0 | 0.0 |
| 17 | 0.0 | 0.0 |
| 18 | 0.0 | 0.0 |
| 19 | 0.0 | 0.0 |
| 20 | 1.0 | 0.0 |
| 21 | 1.0 | 0.0 |
| 22 | 0.0 | 1.0 |
| 23 | 0.0 | 0.0 |
| 24 | 0.0 | 0.0 |
| 25 | 0.0 | 0.0 |
| 26 | 0.0 | 0.0 |
| 27 | 0.0 | 0.0 |
| 28 | 0.0 | 0.0 |
| 29 | 1.0 | 0.0 |
| 30 | 0.0 | 0.0 |
ABPC/SBT
PIPC/TAZ

## Slide 2
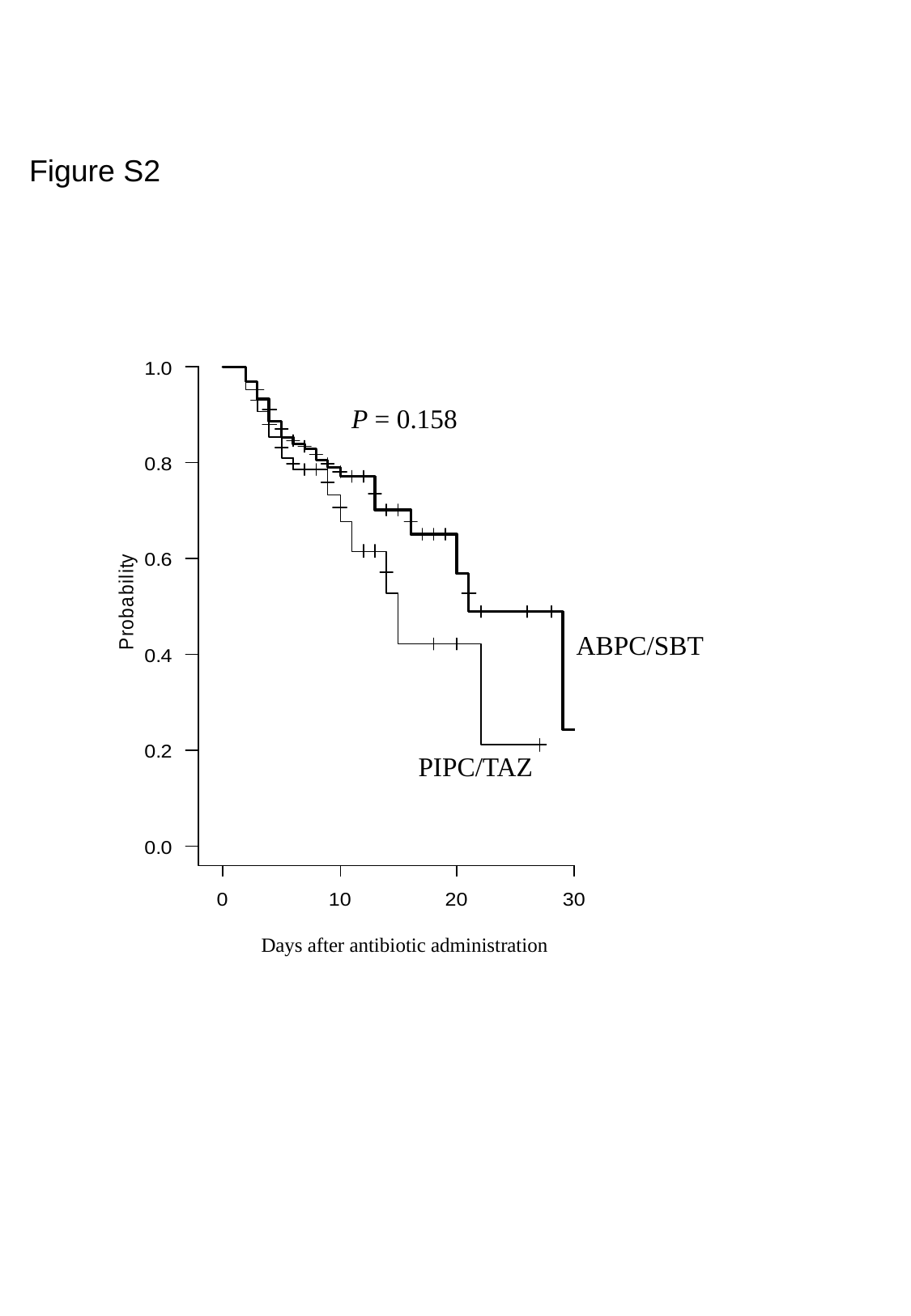

Figure S2
P = 0.158
ABPC/SBT
PIPC/TAZ
Days after antibiotic administration
