# Supplementary material for: Ampicillin sulbactam impacts serum potassium level comparable to piperacillin tazobactam
Source: Sci Rep. 2025 Oct 10;15:35517. doi: 10.1038/s41598-025-19484-8 (PMC12514168; doi:10.1038/s41598-025-19484-8)
Supplement: Supplementary file 2 — Supplementary Information 2. [file 41598_2025_19484_MOESM2_ESM.docx]

**Supplementary Figure Legends**

**Fig. S1** Distribution of duration to onset of hypokalemia from ABPC/SBT or PIPC/TAZ initiation.

The total number of patients who developed hypokalemia was 57 (40 in ABPC/SBT cohort, 17 in PIPC/TAZ cohort). Closed bar denotes the ABPC/SBT cohort, and open bar represents the PIPC/TAZ cohort. ABPC/SBT, Ampicillin/sulbactam; PIPC/TAZ, piperacillin/tazobactam.

**Fig. S2** Kaplan-Meier survival curves of the ABPC/SBT cohort and the PIPC/TAZ cohort for the onset of hypokalemia.

The total number of patients who developed hypokalemia was 57 (40 in ABPC/SBT cohort, 17 in PIPC/TAZ cohort). The horizontal axis shows days after antibiotic administration. The vertical axis shows the proportion of patients without hypokalemia onset. Censoring marks indicate the patients who were finished administering ABPC/SBT or PIPC/TAZ. ABPC/SBT, Ampicillin/sulbactam; PIPC/TAZ, piperacillin/tazobactam.
